# Factors associated with geriatric morbidity and impairment in a megacity of Pakistan

**DOI:** 10.1371/journal.pone.0218872

**Published:** 2019-06-27

**Authors:** Saniya R. Sabzwari, Romaina Iqbal, Zafar Fatmi, Iqbal Azam

**Affiliations:** 1 Department of Family Medicine, Aga Khan University, Karachi, Pakistan; 2 Department of Community Health Sciences, Aga Khan University, Karachi, Pakistan; Nathan S Kline Institute, UNITED STATES

## Abstract

**Background:**

The elderly population is increasing globally. In Pakistan, the elderly comprise 6% of the population that is predicted to triple by 2050. Non-communicable diseases are common health problems of the elderly in Pakistan, however, resulting geriatric impairments and disability are unknown. This study was conducted to determine geriatric impairments and identify associated socio-demographic factors and comorbidities among community dwelling elderly in Karachi, Pakistan.

**Methods:**

A cross-sectional study was conducted during 2013–2014. Community clusters were selected from all sub-districts of Karachi, the largest city of Pakistan. Data was collected from systematically selected households within these clusters from individuals, aged ≥60 years, using standardized questionnaires. Geriatric impairment was assessed through validated questions and tools. We screened for depression, dementia, mobility and functional status. Descriptive statistics were computed for socio-demographic factors. We estimated the prevalence and 95% CI for geriatric impairments and comorbidities.

**Results:**

A total of 1200 community-dwelling elderly participated in this study. More than half (n = 663, 55.3%) were females. The average age of the participants was 68.7 (SD = 7.8) years. Two-thirds suffered from chronic illness and the most common impairments were psychological and cognitive. Females were 2.45 times more at risk of developing three or more geriatric impairments. Participants with no formal education had the highest proportion (43.8%) of geriatric impairments. Participants living with more children were more likely to have three or more impairments.

**Conclusion:**

A high burden of non-communicable diseases and associated impairments were identified among elderly in Karachi, Pakistan. High rates of psychological and cognitive impairments require urgent attention for resources and strategic planning in anticipation of a growing geriatric population.

## Introduction

The elderly population is increasing globally. Between now and 2050, the population aged 60 years and older (elderly) will double from 12% (900 million) to 22% (2 billion). Moreover, approximately 125 million people are currently 80 years or older. The pace of ageing is much higher in developing countries. It is predicted that by 2050, 80% of older individuals will be living in low and middle-income countries (LMICs)[1].

Ageing when accompanied with illness poses a significant challenge for provision of health and social services, particularly for LMICs. The elderly in Pakistan comprise 6% of total population and this proportion is predicted to double by 2050 [2]. The medical care and social needs of this cohort remain largely undefined in the local context [3]. Hospital based studies identify non-communicable diseases and illnesses like osteoarthritis to be common health problems of the elderly in Pakistan [4] [5]. However, the true extent of geriatric impairments and resulting disability is unknown and population-based estimates are not available. Moreover, an assessment of relation between disease and disability, a key indicator of well-being of the elderly, has not been done in Pakistan [6] and such reports are scarce in LMICs [7]. Studies on elderly population in India identified morbidity patterns similar to those in Pakistan, however geriatric assessments were not done [8, 9]. Other regional studies looked at individual impairments, but studies on comprehensive geriatric assessment are scarce [10, 11].

Comprehensive geriatric assessment provides information about cognitive, psychological, mobility, visual and hearing[12] domains of elderly health and identifies geriatric impairment that result from co-morbid illnesses. These impairments also identify presence of common geriatric syndromes such as falls, malnutrition and incontinence all of which are associated with poor health outcomes [13].

Geriatric impairments like vision and hearing have high prevalence globally with estimates as high as 60% to 70% [14]. Nonetheless, a recent trend in high-income countries (HICs) suggests a decline in the number of disabled elderly. This decrease in functional disability may possibly be due to better disease management and use of functional aids [15].

Whereas developed countries have health systems and programs in place to cater for this cohort, we in Pakistan first need to develop a baseline understanding of morbidity patterns and their health implications for our elderly population in order to determine key strategies for health provision.

The goal of our study was to determine geriatric impairments and identify associated socio-demographic factors and comorbidities and among community dwelling elderly in Karachi, Pakistan.

## Materials and methods

### Study design and sampling

A cross-sectional study was conducted during 2013–2014 in all sub-districts of Karachi, the largest city of Pakistan. Karachi is a megacity and has a population of over 20 million. It has a multiethnic, diverse socio-economic population and urban lifestyle.

Multistage cluster sampling was done in Karachi to derive a representative sample from the city. Fifteen enumeration blocks (called primary sampling units as clusters) of Pakistan Bureau of Statistics were randomly selected from the city. Within each cluster, households were systematically selected based on eligibility.

Individual/s (age 60 or older) in the selected household was/were included in the study.

### Sample size

The minimum sample required was 1200. The sample was estimated for 5% prevalence of impairment with a precision of 1.5% using 95% confidence interval. Since, it was a cluster sampling we kept the design effect at 1.5.

### Data collection and assessment tools

Data were collected at the household from the individuals using standardized questionnaires. Study objectives were explained in detail to participants (or their primary caregivers) and written consent was obtained. Information about demographic factors, education level, household, health history, and medication use was recorded in face-to-face interviews. Next of kin were interviewed where participants were unable to respond. Data were collected by research assistants, who were trained to ensure standardized methods of data collection. The research assistant made two attempts to recruit households that were initially unavailable.

Geriatric impairment was assessed through screening questions and validated tools. We screened for depression, dementia, mobility and functional status. Two screening questions were used for depression (low mood and anhedonia) [16] [17] and the 6-CIT (cognitive impairment test) [18–20] was administered. A positive depression screen was identified if either low mood or anhedonia was reported. The 6-CIT assessed memory, attention and orientation. Scoring was done for each item: an incorrect response was given a score of two with a maximum of 10 points. All subjects who scored 8 or more were considered positive for dementia screening [21].

Gait was assessed using the Timed Get Up and Go Test. Completion time of 20 seconds or greater was considered as abnormal [22]. Participants used their ambulatory aid when applicable. Mobility testing was not performed on bed-bound participants.

Functional capacity was assessed by activities of daily living (ADLs) and instrumental activities of daily living (IADLs). ADLs were measured using self-reporting of the Katz ADLs[23] in which independence vs. dependence in five activities was assessed (eating, toileting, bathing, dressing, and ambulating across a room). IADLs were assessed by asking independence in culturally contextual activities (arranging transport, handling money, taking medication, shopping and using the telephone).

The primary outcome was identification of frequency and type of geriatric impairments in the elderly population residing in Karachi. We also studied the association of co-morbid illnesses and socio-economic factors with geriatric impairments.

### Analysis

SPSS version 19 was used for data analysis (IBM SPSS Statistics for Windows, Armonk, NY: IBM Corp). Descriptive statistics were computed for socio-demographic factors. We estimated the prevalence and 95% CI for geriatric impairments and comorbidities. Continuous variables were reported as means (SD) and categorical variables as percentages. We used univariate and multivariate multinomial logistic regression to assess the relationship of socio-economic and demographic characteristics and comorbidities with geriatric impairment.

### Ethical considerations

Ethical review and approval was obtained prior to start of the study from the Ethical Review Committee of the Aga Khan University, Karachi (ID: 1862-FM-ERC-11).

All study participants were recruited after informed written consent. For elderly subjects unable to give consent, the primary caregiver was asked for consent. Interviewers were trained to consider the comfort of the elderly and they took breaks between interviews when required. Results of all medical findings and tests conducted as part of the study were shared with the participant/caregiver. Appropriate referrals were provided as necessary.

All patient data was kept confidential, entered after coding and shared among investigators only for analysis.

## Results

A total of 1200 community-dwelling adults age 60 and above participated in this study. More than half 55.3% (n = 663) were females. The average age of the participants was 68.7 years (SD = 7.8 years). More than half of the participants were uneducated (55.3%) and more than one-third (35.2%) were in the overweight and obese category as shown in Table 1.

**Table 1:** Socio-demographic characteristics of elderly (≥60 years) in Karachi, Pakistan.

	Frequency	Percent
**Patient Age Group (in years)**		
60–64	393	32.8%
65–69	272	22.7%
70–74	261	21.8%
75–84	214	17.8%
85 & above	60	5.0%
**Gender**		
Female	663	55.3%
Male	537	44.8%
**Marital Status**		
Married	663	55.3%
Single	17	1.4%
Widowed	518	43.2%
Divorced	1	.1%
Separated	1	.1%
**Patient Education Level**		
Illiterate	640	53.3%
Below Primary	115	9.6%
Primary	130	10.8%
Middle	70	5.8%
Matric	115	9.6%
Intermediate	40	3.3%
Graduate	53	4.4%
Post Graduate & above	37	3.1%
**Ethnicity**		
Urdu	674	56.2%
Sindhi	60	5.0%
Saraiki	15	1.3%
Punjabi	198	16.5%
Pashto	63	5.3%
Hindku	56	4.7%
Others	134	11.2%
**Body Mass Index**		
<18.5 (underweight)	177	15.4%
18.5–24.99 (Normal)	570	49.4%
25–29.99 (overweight)	280	24.3%
30 & above (obese)	126	10.9%
**Household Size**		
<5	159	13.3%
5–7	341	28.4%
8–9	247	20.6%
10 & above	453	37.8%
**Number of Children in the HH**		
None	178	14.8%
1–2	210	17.5%
3–4	251	20.9%
5–6	259	21.6%
7 & above	302	25.2%

**Fig 1:**
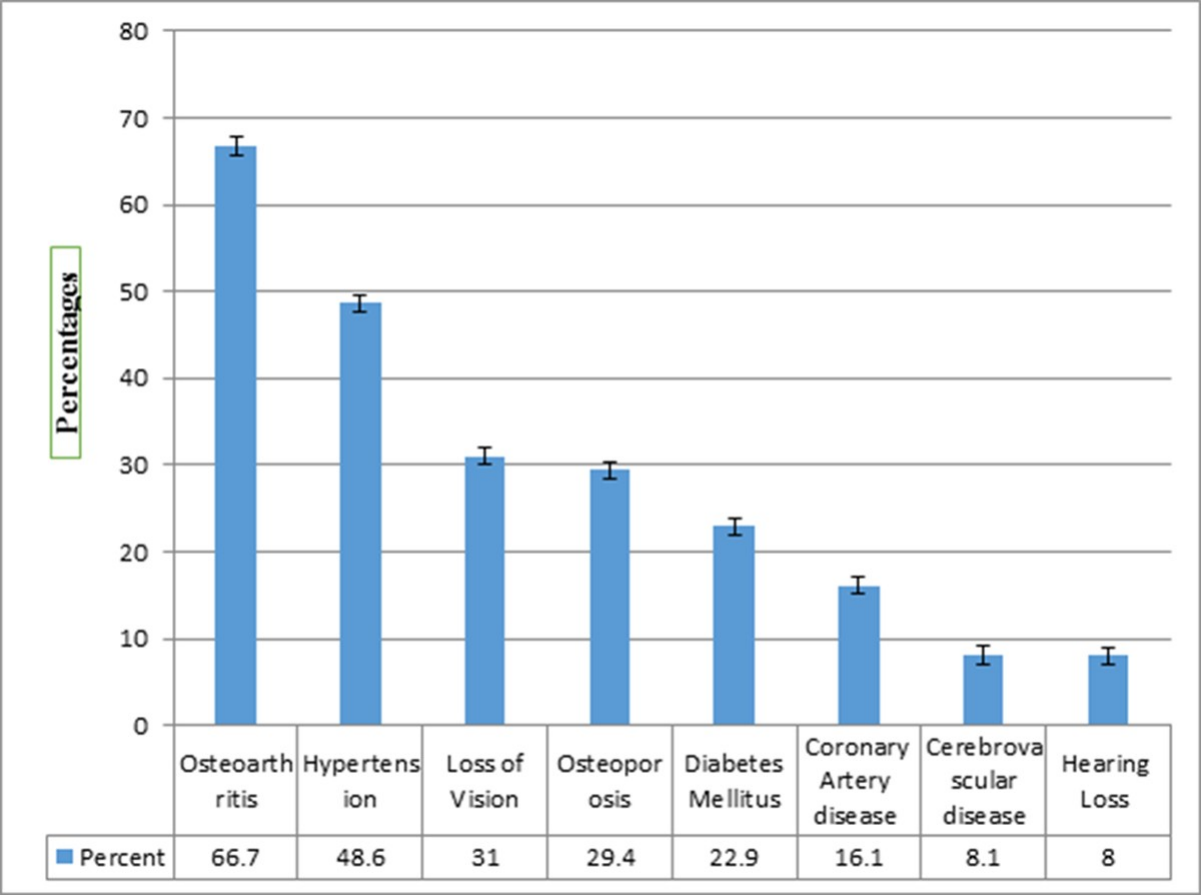
Co morbid illnesses among elderly (≥60 years) in Karachi, Pakistan.

Approximately two thirds (60%) had at least one chronic condition such as diabetes, hypertension, osteoarthritis etc. Half of the participants (51.8%) screened positive for depression, and more than half (63.1%) were cognitively impaired. Visual loss was also reported by 31% participants, and 8% had hearing loss (Fig 1).

Mobility was impaired in almost one-third of the participants (31.1%) either due to pain, instability, or difficulty rising (components of the Get Up and Go Test). One-tenth of the patients had impaired IADLs. Almost all participants were independent in their ADLs. About half of these participants did daily chores at home and almost 10% were still working outside of their homes.

**Fig 2:**
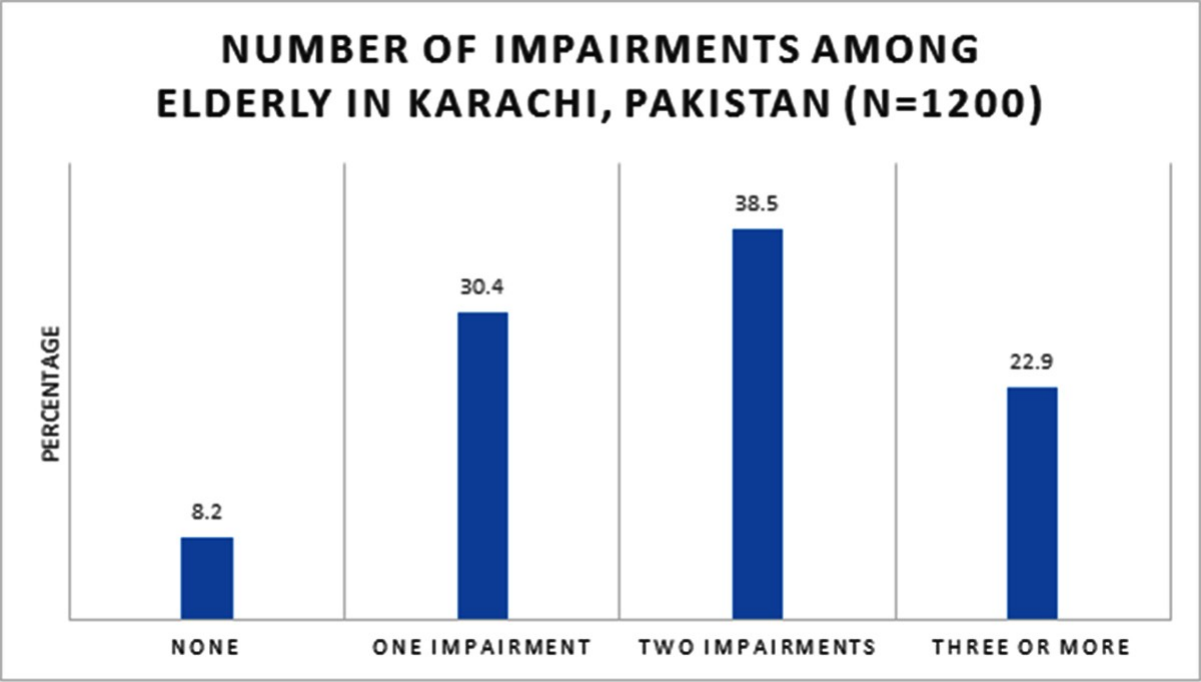
Impairments among elderly (≥60 years) in Karachi, Pakistan.

The study participants were then further categorized as having no impairment, exactly one, exactly two, and exactly three or more impairments. The results reveal that 8.2% of the participants did not have any impairment while 30.4% had one, 38.5% had two, and 22.9% had exactly three or more impairments (Fig 2).

The most common impairments found were psychological and cognitive Table 2.

**Table 2:** Psychological and cognitive symptoms among elderly (≥60 years) in Karachi, Pakistan.

Psychological and Cognitive related Factors n = 1200	Frequency	Percent	95% CI
**Loss of Interest (15 missing)**	762	64.3%	(61.5–67)
**Depressed (5 missing)**	614	51.4%	(48.5–54.2)
**Psychological Impairment (4 missing)**	967	80.9%	78.5–82.9)
**Memory Loss (3 missing)**	623	45.8%	(42.9–48.6)
**Cognitive Impairment (213 missing)**	623	63.1%	(60.1–66.1)

The results of the multinomial regression analysis for factors associated with geriatric impairments are shown in Table 3. About 33.9% males and 42.2% of the females had exactly two geriatric impairments. Among married participants, more than one-third (36.8%) of the participants had one impairment. However, greater of number of impairments were found in those that were unmarried.

**Table 3:** Univariate and multivariate multinomial logistic regression for factors associated with geriatric impairment among elderly (≥60 years) in Karachi, Pakistan.

	Univariate Analysis (Crude)				Multivariable Analysis (Adjusted)			
				p-value				p-value
	Exactly one	Exactly two	Three or more		Exactly one	Exactly two	Three or more	
	OR (95% CI)	OR (95% CI)	OR (95% CI)		OR (95% CI)	OR (95% CI)	OR (95% CI)	
**Patient Age Group (in years)**				<0.001				0.004
60–64	1.45 (0.37, 5.74)	0.44 (0.13, 1.54)	0.29 (0.08, 1.03)		2.21 (0.52, 9.44)	0.85 (0.23, 3.17)	0.60 (0.16, 2.28)	
65–69	1.29 (0.32, 5.17)	0.43 (0.12, 1.52)	0.21 (0.06, 0.75)		1.81 (0.42, 7.80)	0.67 (0.18, 2.53)	0.30 (0.08, 1.19)	
70–74	1.74 (0.42, 7.26)	0.65 (0.18, 2.40)	0.53 (0.14, 1.97)		2.20 (0.50, 9.71)	0.91 (0.24, 3.48)	0.73 (0.18, 2.87)	
75–84	1.15 (0.27, 4.95)	0.81 (0.22, 3.02)	0.55 (0.14, 2.09)		1.47 (0.32, 6.72)	1.16 (0.29, 4.57)	0.76 (0.19, 3.10)	
85 & above (Ref.)								
**Gender**				<0.001				<0.001
Female	1.14 (0.73, 1.80)	2.33 (1.49, 3.63)	3.22 (1.99, 5.18)		1.05 (0.63, 1.75)	1.94 (1.17, 3.21)	2.45 (1.42, 4.25)	
Male (Ref.)								
**Patient Ethnicity**				0.061				
Urdu (Ref.)								
Sindhi	1.14 (0.41, 3.16)	0.94 (0.34, 2.60)	1.50 (0.53, 4.29)					
Punjabi	1.71 (0.83, 3.53)	1.99 (0.98, 4.06)	2.21 (1.05, 4.66)					
Pashto	0.62 (0.23, 1.69)	1.15 (0.46, 2.89)	1.25 (0.47, 3.36)					
Hindko	0.86 (0.33, 2.25)	0.74 (0.28, 1.94)	1.10 (0.40, 2.99)					
Other	1.18 (0.52, 2.67)	1.85 (0.84, 4.06)	2.82 (1.26, 6.32)					
**Patient Marital Status**				<0.001				
Others	0.93 (0.58, 1.49)	1.79 (1.14, 2.81)	2.50 (1.55, 4.05)					
Currently Married (Ref.)								
**Patient Education Level**				<0.001				<0.001
Illiterate	0.91 (0.37, 2.26)	9.12 (2.77, 30.02)	29.79 (3.57, 248.55)		0.92 (0.34, 2.46)	6.98 (1.85, 26.34)	15.08 (1.72, 132.43)	
Below Primary	1.10 (0.36, 3.37)	6.69 (1.73, 25.91)	22.56 (2.44, 208.67)		1.21 (0.36, 4.03)	5.05 (1.13, 22.66)	11.57 (1.17, 114.60)	
Primary	0.90 (0.31, 2.61)	5.48 (1.48, 20.34)	19.83 (2.21, 178.32)		1.00 (0.33, 3.04)	5.05 (1.21, 21.07)	12.40 (1.32, 116.85)	
Middle	2.70 (0.71, 10.22)	8.05 (1.69, 38.44)	10.50 (0.91, 121.39)		2.84 (0.72, 11.18)	6.92 (1.31, 36.55)	6.94 (0.57, 83.83)	
Matric	1.58 (0.54, 4.63)	4.90 (1.28, 18.82)	11.20 (1.19, 105.13)		1.92 (0.62, 5.98)	5.45 (1.26, 23.68)	8.52 (0.86, 84.99)	
Intermediate	0.75 (0.22, 2.52)	2.20 (0.50, 9.75)	4.00 (0.35, 45.38)		1.11 (0.31, 4.05)	2.48 (0.48, 12.89)	5.00 (0.41, 60.69)	
Graduate	1.31 (0.39, 4.45)	4.20 (0.96, 18.33)	2.33 (0.17, 32.58)		1.70 (0.48, 5.98)	4.65 (0.95, 22.73)	2.54 (0.17, 37.06)	
Post Graduate & above (Ref.)								
**Patient Body Mass Index**				<0.001				0.001
<18.5 (underweight)	1.24 (0.52, 2.98)	2.04 (0.88, 4.72)	2.69 (1.14, 6.36)		1.40 (0.57, 3.44)	1.88 (0.79, 4.48)	2.30 (0.94, 5.61)	
18.5–24.99 (Normal) (Ref.)								
25–29.99 (overweight)	0.62 (0.37, 1.04)	0.52 (0.31, 0.86)	0.46 (0.26, 0.80)		0.63 (0.37, 1.06)	0.50 (0.30, 0.85)	0.44 (0.24, 0.78)	
30 & above (obese)	1.54 (0.68, 3.49)	0.93 (0.40, 2.12)	1.12 (0.47, 2.65)		1.63 (0.70, 3.77)	0.84 (0.36, 1.99)	0.91 (0.37, 2.25)	
**Diabetes Mellitus**				0.225				
No (Ref.)								
Yes	1.21 (0.71, 2.08)	1.11 (0.65, 1.88)	0.83 (0.47, 1.47)					
**CAD**				0.441				
No (Ref.)								
Yes	1.61 (0.83, 3.12)	1.30 (0.68, 2.51)	1.34 (0.68, 2.66)					
**Hypertension**				0.127				
No (Ref.)								
Yes	0.80 (0.51, 1.25)	0.99 (0.64, 1.54)	1.17 (0.73, 1.86)					
**CVA**				0.001				0.014
No (Ref.)								
Yes	6.22 (0.83, 46.74)	10.47 (1.43, 76.88)	11.44 (1.54, 85.1)		4.88 (0.64, 37.45)	7.91 (1.06, 59.24)	8.94 (1.17, 68.33)	
**Osteoporosis**				0.062				0.033
No (Ref.)								
Yes	1.89 (1.10, 3.23)	1.48 (0.87, 2.52)	1.81 (1.04, 3.14)		1.98 (1.10, 3.57)	1.30 (0.72, 2.34)	1.57 (0.84, 2.94)	
**Osteoarthritis**				0.004				
No (Ref.)								
Yes	1.11 (0.71, 1.76)	1.30 (0.83, 2.03)	1.97 (1.21, 3.21)					
**Loss of vision**				<0.001				
No (Ref.)								
Yes	13216403.1 (8320572.4, 20992943.9)	46654394.7 (32559487.9, 66850945.3)	519988222.4 (519988222.4, 519988222.4)					
**Hearing Loss**				<0.001				
No (Ref.)								
Yes	15484200.2 (6168107.7, 38870990.7)	29729664.5 (15833983.1, 55820000.9)	449357811.2 (449357811.2, 449357811.2)					
**Household Size**				0.473				
<5 (Ref.)								
5–7	0.89 (0.40, 1.97)	0.79 (0.36, 1.88)	0.48 (0.21, 1.08)					
8–9	0.77 (0.33, 1.79)	0.83 (0.36, 1.88)	0.56 (0.24, 1.30)					
10 & above	0.77 (0.35, 1.66)	0.85 (0.40, 1.81)	0.57 (0.26, 1.24)					
**Total Children in the household**				<0.001				0.004
None (Ref.)								
1–2	1.05 (0.52, 2.10)	1.10 (0.55, 2.21)	1.49 (0.67, 3.28)		1.05 (0.50, 2.20)	1.03 (0.48, 2.21)	1.56 (0.65, 3.73)	
3–4	0.96 (0.49, 1.86)	1.07 (0.55, 2.08)	1.54 (0.72, 3.27)		0.94 (0.45, 1.96)	1.06 (0.50, 2.24)	1.62 (0.69, 3.81)	
5–6	1.32 (0.62, 2.84)	2.39 (1.13, 5.06)	4.23 (1.86, 9.60)		1.39 (0.61, 3.18)	2.25 (0.98, 5.15)	4.37 (1.74, 11.01)	
7 & above	1.19 (0.58, 2.47)	2.35 (1.15, 4.80)	4.09 (1.87, 8.97)		1.25 (0.56, 2.79)	2.10 (0.95, 4.67)	4.15 (1.70, 10.13)	

There was a gradual increase in the number of impairments from ages 60 to 85 years and older. (OR 0.60, 95% CI: 0.16–2.28). Females were 2.45 times (OR: 2.45; 95% CI: 1.42–4.25) more at risk of developing three or more geriatric impairment as compared to males.

Educational level of participants was also significantly associated with geriatric impairment. Participants with no formal education had the highest proportion (43.8%) of geriatric impairments (two), whereas, the majority of participants with at least middle school education had only one geriatric impairment (p-<0.001).

Participants who lived in houses where there were more children were more likely to have 3 or more impairments.

Among patients with co-morbid conditions, those with CVA (OR: 8.94; 95% CI: 1.17–68.33) were more likely to have three or more geriatric impairments. Musculoskeletal problems were found to be significantly associated with a higher number of impairments.

Forty percent of diabetics and 39% of hypertensive patients had exactly two geriatric impairments.

## Discussion

This study is the first of its kind to comprehensively assess a large cohort of community-dwelling elderly individuals in Karachi.

It identified common diseases prevalent among the elderly population and impairments unique to this age group. Association between impairments, co-morbid illnesses and socioeconomic factors were also explored.

South Asians have high rates of hypertension and diabetes with estimates of up to 33% and 12% respectively [24, 25]. In addition, this study confirmed the high disease burden of non-communicable diseases among elderly population. Diabetes and hypertension, due to their chronic nature, have a greater likelihood of disease progression and complications in the elderly, possibly explaining the high number of participants reporting vision loss, cerebrovascular events, and cognitive decline.

Musculoskeletal conditions like osteoarthritis and osteoporosis were frequent. Although self-reported, these may represent true prevalence. Firstly, high rates of obesity were found in this cohort. Obesity as a risk factor for degenerative joint disease (especially weight-bearing joints), has been cited in an earlier study [26]. Secondly, high rates of osteoporosis and the rising incidence of resulting fractures in Asia have also been cited in earlier reports [27]. The poor health outcomes and future dependency resulting from osteoporotic fractures in the elderly are well documented [28,29] and, therefore, require development of programs for community screening and rehabilitation.

There were surprisingly high rates of participants screening positive for depression and cognitive decline. This may be a consequence of stressful life in urban communities [30]. Previous studies cite rates of 3% to 7% for dementia [31, 32] and 10 to 20% for depression [33]. A local study also cited high rates of depression in older individuals [34]. The high numbers found in this study may be a result of converging socio-economic and environmental factors and disease; therefore need further exploration. Moreover, depression and dementia are also known to have a complex association, where depression may be an early indicator of cognitive decline [35].

A multifaceted relationship exists between non-communicable diseases, depression, and dementia. Diabetes has been associated with depression [36] both as its cause and effect [37]. Cognitive impairment has been cited as a complication of long-standing diabetes and hypertension rendering them both risk factors for dementia [38]. Microvascular complications in diabetes result in cerebrovascular disease via white matter changes that independently increase the risk of disability in the elderly [39].

The unprecedented cognitive decline found in this study may well lend credence to the fact that almost two-thirds of dementia patients reside in developing countries [40] making it imperative to focus on this disease and its contributors.

This study also highlighted impairments and common associated factors. Not surprisingly, more impairments were found in females, who due to longer lifespans have greater chances of dependence and functional decline. Other reasons for greater impairment in women include lower muscle and bone mass increasing risk of frailty [41] and gender disparities in healthcare and access for older women which have been reported previously[42] and are likely to be greater in developing countries, requiring further study.

Among socio-economic factors, education level was found to be inversely related to the number of impairments. Poor educational level has not only been associated with poor quality of life scores, [43] but also with a greater number of co-morbid illnesses [44]. A lack of education is a proxy indicator of being socioeconomically disadvantaged. This is likely to result in poor disease awareness and lesser access to health care resulting in more complicated illness, thus greater impairment.

Moreover, education has also been cited as a protective factor for illnesses like dementia [45]. Lower education levels of participants may be another reason for the high rates of cognitive impairment found in this study.

Participants living with more children were found to have a greater number of impairments. This may reflect cultural norms of our country where elderly parents or in-laws, especially those that are ill, often move in with relatives for greater family support.

Another factor significantly associated with a number of impairments was participant weight. Whereas a low BMI may enhance the risk of impairment and mortality in this age group [46], obesity also acts as an independent risk factor for functional decline and disability in the older individual [47].

Diseases affecting mobility like osteoarthritis and osteoporosis were also associated with a greater number of impairments. Early detection and treatment of these conditions may be key to reducing impairment in this age group.

Despite the high rates of cognitive and psychological impairment, low rates of functional decline were found in this study. One possible explanation may be a better communal integration of the elderly individual in our society. Strong social relationships have been cited as a protective factor for functional decline [48, 49].

### Strengths and limitations

This was the first community based study that looked at a geographically representative sample of elderly individuals living in Karachi. The study was adequately powered to assess the primary outcome of geriatric impairments.

A potential weakness of this study that co-morbid illnesses were either self-reported or by caregivers, the possibility of error in disease reporting may have occurred. Every effort, however, was made to utilize standardized and validated assessment tools for confirming psychological, cognitive and gait-related issues reported by participants to minimize self-reporting errors.

## Conclusion

This study provides an initial understanding of the disease status of elderly residing in an urban community of Pakistan. It identified a heavy burden of non-communicable diseases and associated impairments. High rates of psychological and cognitive impairments warrant more in-depth study of the elderly population in rural and semi-urban areas of Pakistan as well.

## Supporting information

S1 Dataset Geriatric impairment data. (SAV)

## References

[1] World Health Organization. Ageing and Health. 2018. Available at: https://www.who.int/news-room/fact-sheets/detail/ageing-and-health. Accessed Sep 2018

[2] World Health Organization. World report on ageing and health 2015. Available at: https://www.who.int/mediacentre/news/releases/2015/older-persons-day/en/. Accessed Sep 2018

[3] Sabzwari SR, Azhar G. Ageing in Pakistan—a new challenge. Ageing International. 2011;36(4):423–7.

[4] Zafar SN, Ganatra HA, Tehseen S, Qidwai W. Health and needs assessment of geriatric patients: results of a survey at a teaching hospital in Karachi. JPMA The Journal of the Pakistan Medical Association. 2006;56(10):470–4. 17144398

[5] Saleem T, Khalid U, Qidwai W. Geriatric patients' expectations of their physicians: findings from a tertiary care hospital in Pakistan. BMC health services research. 2009;9:205 10.1186/1472-6963-9-205 19912619 PMC2780408

[6] Spiers NA, Matthews RJ, Jagger C, Matthews FE, Boult C, Robinson TG, et al Diseases and impairments as risk factors for onset of disability in the older population in England and Wales: findings from the Medical Research Council Cognitive Function and Ageing Study. The journals of gerontology Series A, Biological sciences and medical sciences. 2005;60(2):248–54. 10.1093/gerona/60.2.248 15814870

[7] Gutiérrez-Robledo LM. Looking at the future of geriatric care in developing countries. The Journals of Gerontology Series A: Biological Sciences and Medical Sciences. 2002;57(3):M162–M7. 10.1093/gerona/57.3.m162 11867652

[8] Sehgal RK, Garg R, Anand S, Dhot PS, Singhal P. A study of the morbidity profile of geriatric patients in rural areas of Ghaziabad, Uttar Pradesh. International Journal of Medical Science and Public Health. 2016;5(2):176–81.

[9] Barua K, Borah M, Deka C, Kakati R. Morbidity pattern and health-seeking behavior of elderly in urban slums: A cross-sectional study in Assam, India. Journal of family medicine and primary care. 2017;6(2):345 10.4103/2249-4863.220030 29302545 PMC5749084

[10] Lee JC, Danker AN, Wong YH, Lim MY. Hearing Loss amongst the Elderly in a Southeast Asian Population-A Community-based Study. Annals of the Academy of Medicine, Singapore. 2017;46(4):145–54. 28485462

[11] Munshi YI, Iqbal M, Rafique H, Ahmad Z. Geriatric morbidity pattern and depression in relation to family support in aged population of Kashmir valley. The Internet Journal of Geriatrics and Gerontology. 2008;4(1).

[12] Chaudhry SI, McAvay G, Ning Y, Allore HG, Newman AB, Gill TM. Geriatric impairments and disability: the cardiovascular health study. Journal of the American Geriatrics Society. 2010;58(9):1686–92. 10.1111/j.1532-5415.2010.03022.x 20863328 PMC2946108

[13] Won C, Yoo H, Yu S, Kim C, Dumlao L, Dewiasty E, et al Lists of geriatric syndromes in the Asian-Pacific geriatric societies. European Geriatric Medicine. 2013;4(5):335–8.

[14] Rooth MA. The prevalence and impact of vision and hearing loss in the elderly. North Carolina medical journal. 2017;78(2):118–20. 10.18043/ncm.78.2.118 28420775

[15] Gill TM, Gahbauer EA. Overestimation of chronic disability among elderly persons. Archives of internal medicine. 2005;165(22):2625–30. 10.1001/archinte.165.22.2625 16344420

[16] Li C, Friedman B, Conwell Y, Fiscella K. Validity of the Patient Health Questionnaire 2 (PHQ‐2) in identifying major depression in older people. Journal of the American Geriatrics Society. 2007;55(4):596–602. 10.1111/j.1532-5415.2007.01103.x 17397440

[17] Tsoi KK, Chan JY, Hirai HW, Wong SY. Comparison of diagnostic performance of Two-Question Screen and 15 depression screening instruments for older adults: systematic review and meta-analysis. The British Journal of Psychiatry. 2017:bjp. bp. 116.186932. 10.1192/bjp.bp.116.186932 28209592

[18] Brooke P, Bullock R. Validation of a 6 item cognitive impairment test with a view to primary care usage. International journal of geriatric psychiatry. 1999;14(11):936–40. 10556864

[19] K Upadhyaya A, Rajagopal M, M Gale T. The six item cognitive impairment test (6-CIT) as a screening test for dementia: comparison with mini-mental state examination (MMSE). Current aging science. 2010;3(2):138–42. 20158493 10.2174/1874609811003020138

[20] Sabzwari S, Bhanji S, Nanji K. Choosing a Reliable Cognitive Test for Community Screening of Dementia in Pakistan. Ageing International. 2016;41(2):167–77.

[21] Hessler J, Brönner M, Etgen T, Ander K-H, Förstl H, Poppert H, et al Suitability of the 6CIT as a screening test for dementia in primary care patients. Aging & mental health. 2014;18(4):515–20. 24256425 10.1080/13607863.2013.856864

[22] Shumway-Cook A, Brauer S, Woollacott M. Predicting the probability for falls in community-dwelling older adults using the Timed Up & Go Test. Physical therapy. 2000;80(9):896–903. 10960937

[23] Katz S. Assessing self‐maintenance: activities of daily living, mobility, and instrumental activities of daily living. Journal of the American Geriatrics Society. 1983;31(12):721–7. 6418786 10.1111/j.1532-5415.1983.tb03391.x

[24] Ghaffar A, Reddy KS, Singhi M. Burden of non-communicable diseases in South Asia. BMJ: British Medical Journal. 2004;328(7443):807 10.1136/bmj.328.7443.807 15070638 PMC383378

[25] Ramachandran A, Snehalatha C, Ma RCW. Diabetes in South-East Asia: an update. Diabetes research and clinical practice. 2014;103(2):231–7. 10.1016/j.diabres.2013.11.011 24300015

[26] Iqbal MN, Haidri FR, Motiani B, Mannan A. Frequency of factors associated with knee osteoarthritis. JPMA-Journal of the Pakistan Medical Association. 2011;61(8):786. 22356003

[27] Mithal A, Kaur P. Osteoporosis in Asia: a call to action. Current osteoporosis reports. 2012;10(4):245–7. 10.1007/s11914-012-0114-3 22898971

[28] Roth T, Kammerlander C, Gosch M, Luger T, Blauth M. Outcome in geriatric fracture patients and how it can be improved. Osteoporosis international. 2010;21(4):615–9. 10.1007/s00198-010-1401-4 21058001

[29] Mohd-Tahir N, Li S. Economic burden of osteoporosis-related hip fracture in Asia: a systematic review. Osteoporosis International. 2017;28(7):2035–44. 10.1007/s00198-017-3985-4 28314898

[30] Geronimus AT, Pearson JA, Linnenbringer E, Schulz AJ, Reyes AG, Epel ES, et al Race-ethnicity, poverty, urban stressors, and telomere length in a Detroit community-based sample. Journal of health and social behavior. 2015;56(2):199–224. 10.1177/0022146515582100 25930147 PMC4621968

[31] Kalaria RN, Maestre GE, Arizaga R, Friedland RP, Galasko D, Hall K, et al Alzheimer's disease and vascular dementia in developing countries: prevalence, management, and risk factors. The Lancet Neurology. 2008;7(9):812–26. 10.1016/S1474-4422(08)70169-8 18667359 PMC2860610

[32] Brookmeyer R, Johnson E, Ziegler-Graham K, Arrighi HM. Forecasting the global burden of Alzheimer’s disease. Alzheimer's & dementia: the journal of the Alzheimer's Association. 2007;3(3):186–91. 10.1016/j.jalz.2007.04.381 19595937

[33] Barua A, Ghosh MK, Kar N, Basilio MA. Prevalence of depressive disorders in the elderly. Annals of Saudi medicine. 2011;31(6):620 10.4103/0256-4947.87100 22048509 PMC3221135

[34] Bhamani MA, Karim MS, Khan MM. Depression in the elderly in Karachi, Pakistan: a cross sectional study. BMC psychiatry. 2013;13(1):181. 23819509 10.1186/1471-244X-13-181 PMC3704964

[35] Korczyn AD, Halperin I. Depression and dementia. Journal of the neurological sciences. 2009;283(1–2):139–42. 10.1016/j.jns.2009.02.346 19345960

[36] De Jonge P, Roy J, Saz P, Marcos G, Lobo A. Prevalent and incident depression in community-dwelling elderly persons with diabetes mellitus: results from the ZARADEMP project. Diabetologia. 2006;49(11):2627–33. 10.1007/s00125-006-0442-x 17019601

[37] Nouwen A, Winkley K, Twisk J, Lloyd C, Peyrot M, Ismail K, et al Type 2 diabetes mellitus as a risk factor for the onset of depression: a systematic review and meta-analysis. Springer; 2010. 10.1007/s00125-010-1874-x PMC2974923 20711716

[38] Biessels GJ, Staekenborg S, Brunner E, Brayne C, Scheltens P. Risk of dementia in diabetes mellitus: a systematic review. The Lancet Neurology. 2006;5(1):64–74. 10.1016/S1474-4422(05)70284-2 16361024

[39] Group LS. 2001–2011: a decade of the LADIS (Leukoaraiosis And DISability) Study: what have we learned about white matter changes and small-vessel disease? Cerebrovascular diseases. 2011;32(6):577–88. 10.1159/000334498 22277351

[40] Prince M, Acosta D, Albanese E, Arizaga R, Ferri CP, Guerra M, et al Ageing and dementia in low and middle income countries–Using research to engage with public and policy makers. International review of psychiatry. 2008;20(4):332–43. 10.1080/09540260802094712 18925482 PMC2582830

[41] Frisoli A Jr, Chaves PH, Ingham SJM, Fried LP. Severe osteopenia and osteoporosis, sarcopenia, and frailty status in community-dwelling older women: results from the Women's Health and Aging Study (WHAS) II. Bone. 2011;48(4):952–7. 10.1016/j.bone.2010.12.025 21195216

[42] Cameron KA, Song J, Manheim LM, Dunlop DD. Gender disparities in health and healthcare use among older adults. Journal of Women's Health. 2010;19(9):1643–50. 10.1089/jwh.2009.1701 20695815 PMC2965695

[43] Tajvar M, Arab M, Montazeri A. Determinants of health-related quality of life in elderly in Tehran, Iran. BMC public health. 2008;8(1):323. 18808675 10.1186/1471-2458-8-323 PMC2567978

[44] Marengoni A, Winblad B, Karp A, Fratiglioni L. Prevalence of chronic diseases and multimorbidity among the elderly population in Sweden. American journal of public health. 2008;98(7):1198–200. 10.2105/AJPH.2007.121137 18511722 PMC2424077

[45] Brayne C, Ince PG, Keage HA, McKeith IG, Matthews FE, Polvikoski T, et al Education, the brain and dementia: neuroprotection or compensation? EClipSE Collaborative Members. Brain. 2010;133(8):2210–6. 20826429 10.1093/brain/awq185

[46] MillerSL, WolfeRR. The danger of weight loss in the elderly. The journal of nutrition, health & aging. 2008;12(7):487–91.10.1007/BF0298271018615231

[47] Chen H, Guo X. Obesity and functional disability in elderly Americans. Journal of the American Geriatrics Society. 2008;56(4):689–94. 10.1111/j.1532-5415.2007.01624.x 18266843 PMC2391089

[48] Avlund K, Lund R, Holstein BE, Due P, Sakari-Rantala R, Heikkinen R-L. The impact of structural and functional characteristics of social relations as determinants of functional decline. The Journals of Gerontology Series B: Psychological Sciences and Social Sciences. 2004;59(1):S44–S51. 10.1093/geronb/59.1.s44 14722343

[49] Cramm JM, Van Dijk HM, Nieboer AP. The importance of neighborhood social cohesion and social capital for the well being of older adults in the community. The Gerontologist. 2012;53(1):142–52. 10.1093/geront/gns052 22547088

